# Ultrasonographic pouch-perineum distance integrated into the multimodal diagnostic algorithm enables accurate early classification and surgical planning for anorectal malformation: an 11-year two-center study

**DOI:** 10.1007/s00383-026-06406-6

**Published:** 2026-04-03

**Authors:** Shunya Takada, Hiroo Uchida, Akinari Hinoki, Chiyoe Shirota, Satoshi Makita, Akihiro Yasui, Aitaro Takimoto, Daiki Kato, Takuya Maeda, Hiroki Ishii, Hajime Asai, Kazuki Ota, Takahisa Tainaka

**Affiliations:** 1 Department of Pediatric Surgery, Nagoya University Graduate School of Medicine, 65 Tsurumai-cho, Showa-ku, Nagoya, 466-8550 Japan; 2 Department of Pediatric Surgery, Anjo Kosei Hospital, 28 Higashihirokute, Anjo-cho, Anjo city, Aichi prefecture 446-8602 Japan

**Keywords:** Anorectal malformation, Ultrasonography, Pouch-perineum distance, Diagnostic algorithm

## Abstract

**Purpose:**

Accurate early classification of anorectal malformations (ARM) is essential for selecting the initial surgical strategy. This study evaluated the diagnostic performance of ultrasonographic pouch–perineum distance (PPD) measurement within a multimodal diagnostic algorithm for early ARM classification.

**Methods:**

This two-center retrospective study included 156 patients with ARM who underwent definitive surgery between 2014 and 2024. The algorithm incorporated physical examination, meconium passage assessment, contrast imaging, and ultrasonographic PPD measurement. PPD was evaluated in patients without visible perineal fistulas. Final diagnosis was confirmed by contrast studies and intraoperative findings.

**Results:**

The algorithm correctly classified ARM types in 155 of 156 patients (99.4%). Ultrasonographic PPD measurement was performed in 51 patients without visible perineal fistulas. In this subgroup, median PPD was significantly lower in L-ARM than in NL-ARM (6.5 vs. 15.0 mm, *p* < 0.001). ROC analysis showed excellent discrimination by PPD (AUC = 0.97); a 10-mm cutoff yielded 95.5% sensitivity and 89.7% specificity for distinguishing L-ARM from NL-ARM.

**Conclusion:**

Ultrasonographic PPD measurement is a useful adjunct in the early evaluation of neonatal ARM. When incorporated into a multimodal diagnostic algorithm, it can support classification in patients without visible perineal fistulas and may help guide timely surgical decision-making in selected cases.

## Introduction

Anorectal malformations (ARM) are a spectrum of congenital anomalies that require prompt neonatal diagnosis and accurate preoperative assessment for appropriate surgical planning. Classification into low-type ARM (L-ARM) and non-low-type ARM (NL-ARM) is clinically essential because it guides the choice between primary anoplasty and staged repair with diverting colostomy in the neonatal period. Traditionally, prone cross-table lateral radiography or invertography has been the standard for ARM classification, typically performed 18–24 h after birth to allow adequate rectal gas accumulation. A rectal gas–perineum distance threshold of 10 mm was used to distinguish L-ARM from NL-ARM [1–3].

Recently, ultrasonography has emerged as a reliable, noninvasive, and radiation-free alternative for ARM evaluation [1, 2, 4, 5]. This enables real-time bedside assessment that can be safely repeated, making it ideal for neonates. Among sonographic parameters, the pouch–perineum distance (PPD), which is the distance between the distal rectal pouch and the anal dimple, has been recognized as a key quantitative marker for ARM classification [4–8]. Both suprapubic and perineal ultrasound approaches allow visualization of the distal rectum and fistulous tract. While several studies have demonstrated a strong correlation between ultrasonographic and radiographic findings [6, 9, 10], clinical evidence remains limited, and no consensus has been reached on the optimal PPD cutoff values for classification [7, 8].

At our institutions, we developed a systematic multimodal diagnostic algorithm that integrates ultrasonographic evaluation with physical examination and contrast imaging. This approach has replaced traditional methods in our clinical practice. In patients without a visible perineal fistula, when the multimodal assessment suggests L-ARM, early definitive surgery, such as primary anoplasty, may be considered, which may help avoid unnecessary colostomy in selected cases. Based on this 10-year experience, the present study clarifies the diagnostic role of ultrasonographic PPD measurement within our multimodal diagnostic algorithm. It validates its overall diagnostic accuracy in a large two-center cohort.

## Methods

This study was approved by the Institutional Ethics Board of Nagoya University (approval number: 2025-0021). Anjo Kosei Hospital participated as an information-providing institution and provided de-identified clinical data in accordance with the approved study protocol and applicable institutional procedures. Given the retrospective design and use of anonymized data, the requirement for written informed consent was waived, and an opt-out approach was adopted.

### Data collection

We conducted a retrospective observational study of patients with ARM who underwent definitive surgery between January 1, 2014, and December 31, 2024, at two tertiary pediatric surgical centers in Japan: Nagoya University Hospital and Anjo Kosei Hospital. All clinical data were centralized at Nagoya University, and data transfer from Anjo Kosei was conducted in a de-identified, secure manner, in accordance with institutional and national data protection requirements. Patients were included if their complete clinical and imaging data were available. The exclusion criteria were: (1) absence of ultrasonographic PPD measurement at the initial neonatal evaluation in patients without visible perineal fistulas (e.g., initial colostomy performed at outside hospitals before evaluation at our institutions), and (2) cloacal malformations owing to their distinct anatomy and the inapplicability of PPD assessment. Clinical data, including sex, gestational age, comorbidities, imaging findings, diagnoses, surgical procedures, and intraoperative findings, were collected from the medical records.

### Ultrasonographic evaluation of ARM

**Fig. 1:**
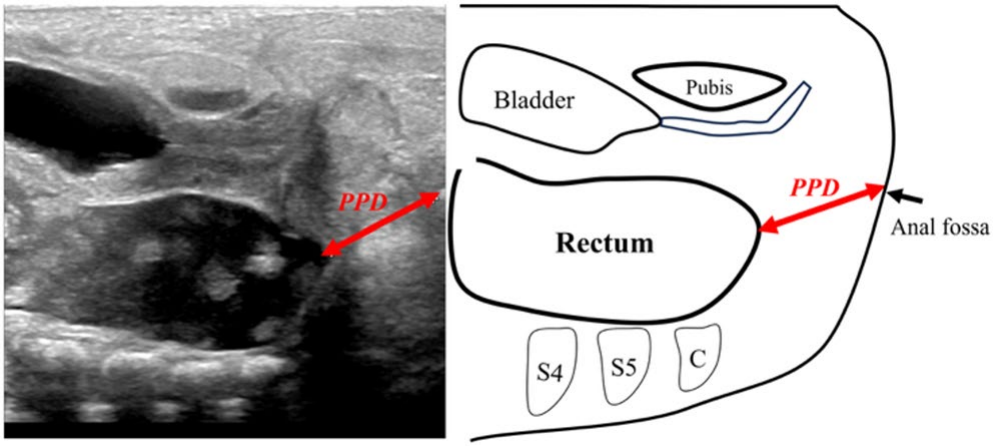
Ultrasonographic view of the suprapubic approach

In patients without visible perineal fistulas, experienced pediatric surgeons performed ultrasonographic evaluation of the ARM to measure the PPD, defined as the distance between the rectal pouch and anal dimple. Ultrasonographic PPD assessment was performed jointly by at least two observers, who reached consensus on the images considered appropriate for measurement. For clinically stable neonates, repeat evaluation was performed on postnatal day 1 when feasible; in such cases, the postnatal day 1 examination was used for decision-making. The median PPD value obtained from the selected images was adopted for analysis to minimize error. Two ultrasonographic approaches were employed:

Suprapubic approach: The probe was placed sagittally on the lower abdomen, just above the pubic symphysis, to visualize the rectal pouch in relation to pelvic structures. Forceps were placed on the anal dimple to indicate the perineal surface and facilitate PPD measurement (Fig. 1).

**Fig. 2:**
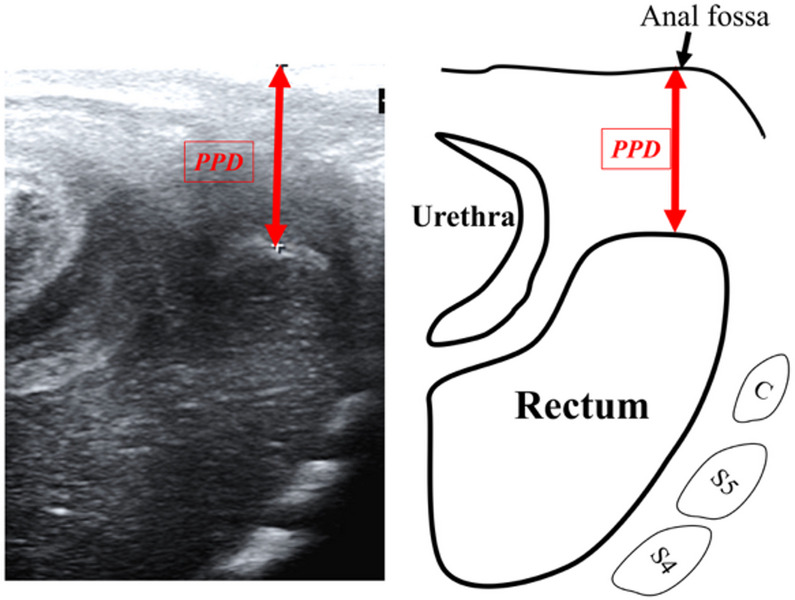
Ultrasonographic view of the perineal approach

Perineal approach: When the rectal pouch could not be clearly identified using the suprapubic approach, an additional perineal scan was performed. The neonate was placed in the supine position, and a high-frequency linear probe was applied sagittally to the perineum with minimal pressure to avoid anatomical distortion (Fig. 2).

### Stepwise multimodal diagnostic algorithm for early neonatal ARM classification

**Fig. 3:**
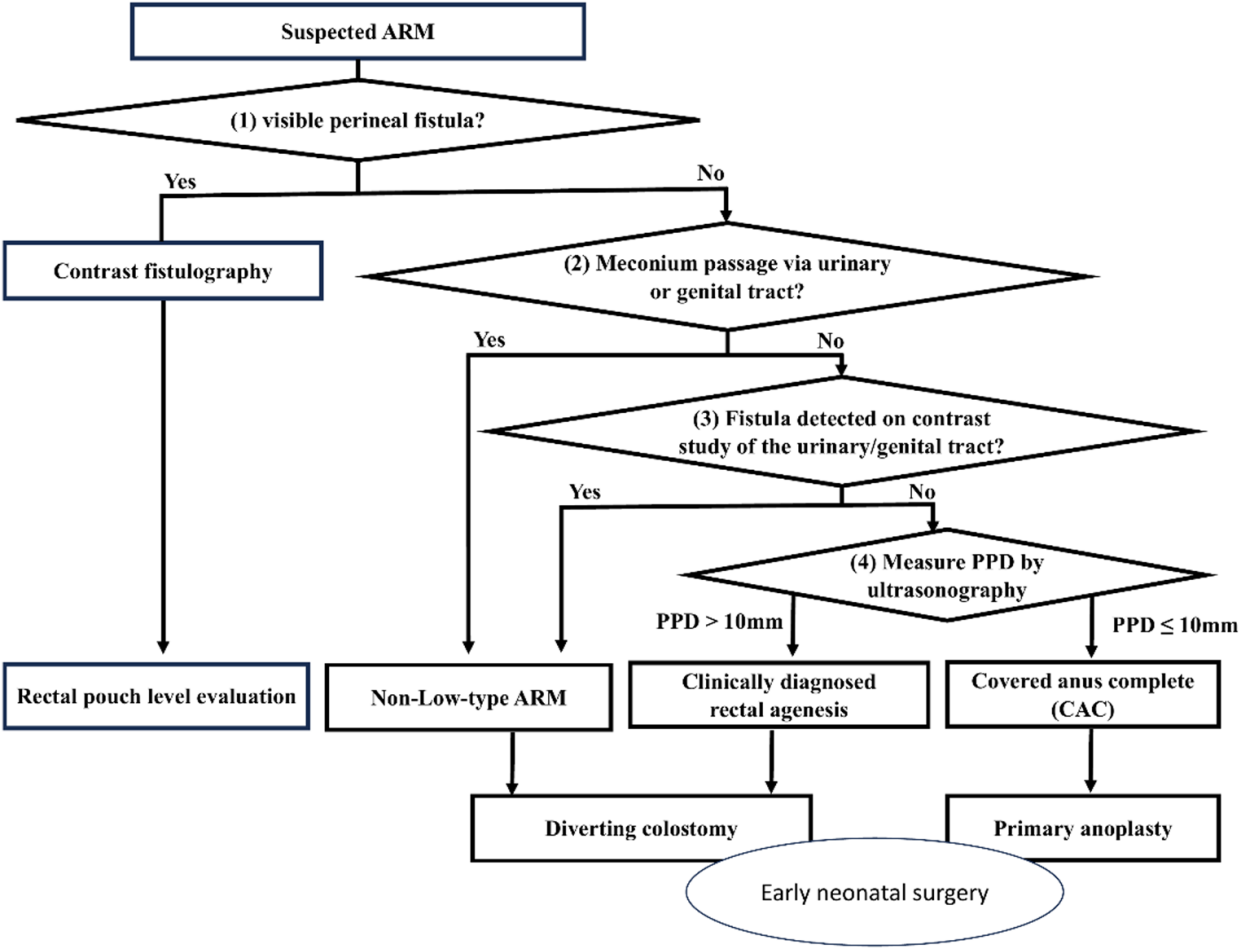
Stepwise multimodal diagnostic algorithm for early neonatal ARM classification and treatment planning. PPD by ultrasonography is used as the next decision criterion (cutoff: 10 mm). *ARM* anorectal malformation;* L-ARM* low anorectal malformation;* NL-ARM* non-low anorectal malformation;* CAC* covered anus complete;* PPD* pouch–perineum distance

To differentiate L-ARM from NL-ARM in the neonatal period, we applied a structured multimodal diagnostic algorithm comprising four components: (1) physical examination; (2) assessment of meconium passage; (3) contrast studies, including fistulography, urethrocystography, and genitography; and (4) ultrasonographic measurement of PPD (Fig. 3). The algorithm proceeds stepwise. First, physical examination assesses the presence of a visible perineal fistula and meconium discharge. Second, meconium passage through the urinary or genital tract is considered definitive evidence of NL-ARM. Third, contrast studies of the urinary and genital tracts are performed to identify rectourethral/rectovesical or rectovaginal fistulas and confirm NL-ARM. In female patients, the distinction between rectovaginal and rectovestibular fistulas was confirmed by review of the electronic medical records, including contrast studies and operative findings. Finally, in patients without demonstrable fistulas on physical examination or contrast studies, ultrasonographic PPD was used as the next decision criterion. A PPD ≤ 10 mm is interpreted as suggestive of L-ARM (specifically covered anus complete [CAC]), whereas a PPD > 10 mm indicates NL-ARM.

### Treatment strategy and definitive diagnosis

For L-ARM cases with a visible perineal fistula, anorectoplasty was scheduled in the late neonatal period after completion of the diagnostic evaluation. In contrast, for CAC, definitive anorectoplasty was performed in the early neonatal period, depending on the infant’s general condition and completion of the diagnostic evaluation. The final diagnosis was determined through direct perineal exploration during definitive surgery.

For NL-ARM cases, diverting colostomy was performed during the early neonatal period. Subtype classification was subsequently determined using contrast studies via the stoma and confirmed intraoperatively during definitive anorectoplasty. In female patients, the fistula opening (vaginal vs. vestibular) was specifically verified on contrast studies and operative findings.

### Statistical analysis

Statistical analyses were performed using Python 3.x. Data normality was assessed using the Shapiro–Wilk test. Group comparisons were performed using the Kruskal–Wallis test and Mann–Whitney U test, with Dunn–Bonferroni post hoc adjustments, when appropriate. Receiver operating characteristic (ROC) curve analysis was used to determine the optimal diagnostic thresholds. All tests were two-sided, with statistical significance set at *p* < 0.05. All analyses and tabulated results were based on final diagnoses confirmed by intraoperative findings.

## Results

### Patient characteristics

During the study period, 171 patients with ARM underwent definitive surgery at the two participating institutions. Fifteen patients were excluded, leaving 156 patients for the final analysis. Among patients without visible perineal fistulas, 10 were excluded owing to absence of ultrasonographic PPD measurement at the initial neonatal evaluation: seven had undergone initial colostomy at outside hospitals before evaluation at our institutions, and three had an inability to perform ultrasonography on admission because of emergent clinical conditions. In addition, five patients with cloacal malformations were excluded owing to their distinct anatomy and the inapplicability of PPD assessment. Consequently, the final cohort comprised 127 patients with L-ARM and 29 with NL-ARM, classified based on the intraoperative diagnosis. The L-ARM group included 105 patients with perineal fistulas and 22 patients with CAC. The NL-ARM group comprised 17 intermediate- and 12 high-type malformations.

Patient characteristics based on the final diagnosis are summarized in Table 1. The L-ARM group had a significantly higher proportion of female patients than the NL-ARM group (69/127 vs. 2/29, *p* < 0.001). There was no significant difference in the median birth weight between the groups (2810 g vs. 2652 g, *p* = 0.16). Prematurity was more frequent in the NL-ARM group (31.0% vs. 15.0%), although the difference was not statistically significant (*p* = 0.059). Chromosomal anomalies were observed in both groups with no significant difference (15.0% vs. 6.9%, *p* = 0.37). In contrast, filar lipoma (20.7% vs. 6.0%, *p* = 0.025) and esophageal atresia/tracheoesophageal fistula (EA/TEF) (34.5% vs. 6.0%, *p* < 0.001) were significantly more common in the NL-ARM group. The NL-ARM subtypes included rectobulbar urethral fistulas (*n* = 16), rectoprostatic urethral fistulas (*n* = 10), true rectovaginal fistulas (*n* = 2; confirmed by contrast studies and operative findings), and anorectal agenesis (*n* = 1).

**Table 1:** Patient characteristics according to the ARM subtype

	L-ARM (*n* = 127)	NL-ARM (*n* = 29)	*p*-value
Sex (M/F)	58/69	27/2	< 0.001
Birth weight (g)	2810 (2416–3157)	2652 (2340–2960)	0.16
Prematurity (%)	19 (15.0%)	9 (31.0%)	0.059
Chromosomal anomaly (%)	19 (15.0%)	2 (6.9%)	0.37
Filar lipoma (%)	8 (6.0%)	6 (20.7%)	0.025
EA/TEF (%)	7 (6%)	10 (34.5%)	< 0.001

Sex distribution is presented as male or female. Birth weight is expressed as the median (interquartile range, IQR). Prematurity, chromosomal anomalies, and esophageal atresia/tracheoesophageal fistulas (EA/TEF) are expressed as percentages

### Diagnostic performance of the multimodal algorithm (overall cohort)

Using our stepwise multimodal diagnostic algorithm, ARM types were classified as follows. Physical examination revealed a perineal fistula in 105 patients diagnosed with an L-ARM. Among the remaining 51 patients without visible fistulas, 10 were diagnosed with NL-ARM (seven intermediate-types and three high-types) based on meconium discharge via the urinary or genital tract. In the 41 patients without evident meconium passage by postnatal day 1, contrast studies revealed rectourinary or rectovaginal fistulas in five cases (four intermediate-type and one high-type). Ultrasonographic PPD measurements were used as the final classification criterion in the remaining 36 patients without demonstrable fistulas on both clinical examination and contrast evaluation. Twenty-one patients with PPD ≤ 10 mm were classified as L-ARM (all with CAC), whereas 15 patients with PPD > 10 mm were classified as NL-ARM and underwent colostomy. Among these 15 cases, postoperative contrast studies via colostomy confirmed six intermediate-type cases, eight high-type malformations, and one CAC case, the latter of which represented a misclassification. This misclassified case had a PPD of 13 mm on postnatal day 0 and underwent unnecessary colostomy (details are described in the subgroup analysis below). Overall, this multimodal diagnostic algorithm correctly identified the final diagnosis in 155 of the 156 patients (99.4% accuracy).

**Table 2:** Ultrasonographic pouch–perineum distance (PPD) evaluation by final diagnosis

	CAC (*n* = 22)	NL-ARM (*n* = 29)	*p*-value
Postnatal day of evaluation (day)	0 (0–1)	0 (0–1)	
PPD (mm)	6.5 (3.5–7.6)	15.0 (14.0–19.0)	*p* < 0.001

All the classifications in this table are based on the final diagnosis. Values are expressed as median (interquartile range)

*CAC* covered anus complete; *NL-ARM* non-low-type anorectal malformation; *PPD* pouch–perineum distance

### Focused subgroup analysis of patients without a perineal fistula

**Fig. 4:**
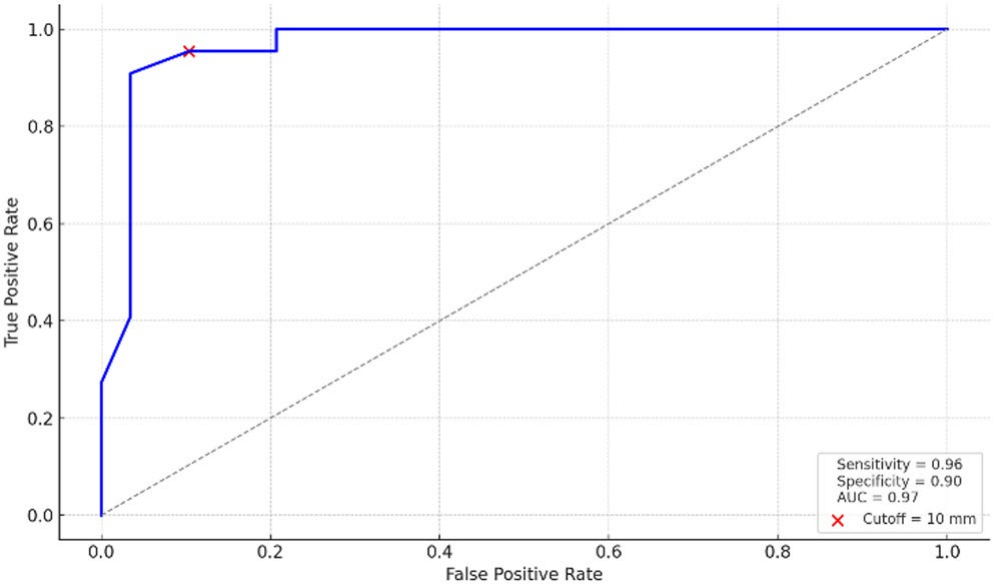
Receiver operating characteristic curve of pouch–perineum distance for differentiating L-ARM vs. NL-ARM

Within the multimodal algorithm, PPD served as the final classification criterion in 36 patients who had no clinical or contrast evidence of a fistula. For subgroup analysis, however, PPD measurements from all 51 patients without visible perineal fistulas were evaluated, including 29 with NL-ARM and 22 with L-ARM classified as CAC (Table 2). The median timing of the ultrasonographic evaluation was postnatal day 0 (IQR, 0–1). The median PPD differed significantly between groups—6.5 mm (IQR, 3.5–7.6) for the L-ARM versus 15.0 mm (IQR, 14.0–19.0) for the NL-ARM group (*p* < 0.001), with no overlap in the interquartile ranges. ROC analysis demonstrated excellent discriminative ability (AUC = 0.97) and identified 10 mm as the optimal cut-off, yielding 95.5% sensitivity (21/22) and 89.7% specificity (26/29) (Fig. 4).

Three NL-ARM cases with PPD ≤ 10 mm were correctly classified as NL-ARM based on clinical evidence of meconium discharge from the urinary tract, despite the low PPD values suggesting L-ARM. In contrast, one L-ARM patient with a PPD of 13.0 mm on postnatal day 0 was initially misdiagnosed as NL-ARM and underwent unnecessary colostomy. The patient was born late at night, and an ultrasound was performed the following morning, with insufficient time for rectal pouch descent. Additionally, no preoperative PPD reevaluation was performed in the operating room. The patient was definitively diagnosed with CAC after a contrast study using a colostomy. When integrating the PPD evaluation with other findings, our multimodal diagnostic approach correctly identified the final diagnosis in 50 of 51 cases (98.0% accuracy) in this subgroup.

## Discussion

Accurate diagnosis of anorectal malformations during the neonatal period is crucial, as it directly determines the initial surgical strategy. Historically, radiographic modalities, such as prone cross-table lateral imaging or invertography, have been widely employed for ARM classification; however, their diagnostic accuracy varies considerably, with reported sensitivities ranging from 70% to 95% and specificities ranging from 67% to 100% [3]. In recent years, ultrasonography has emerged as a promising radiation-free alternative for ARM evaluation [1, 2, 4, 5]. Ultrasonography remains advantageous as a first-line modality in many neonatal intensive care settings because it is safe, repeatable, and feasible at the bedside. Among ultrasonographic parameters, PPD has been reported as a quantitative, objective marker for distinguishing L-ARM from NL-ARM. Previous studies have reported PPD thresholds ranging from 5 to 15 mm [4, 5]. This variation is attributable to differences in measurement techniques, assessment timing, and heterogeneous patient populations. Hosokawa et al. demonstrated that PPD decreases from postnatal day 0 to day 1 due to the physiological descent of the distal rectal pouch and proposed a 9 mm cut-off with an AUC of 0.864 [7]. Ultrasonography has also shown utility in detecting fistulas at the day of birth [6], and its diagnostic performance is comparable to that of contrast imaging for fistula detection [10]. Nevertheless, no consensus has been reached regarding its optimal role in clinical decision-making [8, 9].

The present study highlights that PPD is the most reliable metric when interpreted within a multimodal diagnostic pathway, rather than as an isolated metric. In this study, multimodal assessment, including physical examination, evaluation of meconium passage, contrast imaging, and PPD, enabled the accurate differentiation of ARM subtypes in 99.4% of patients.

This accuracy met or exceeded the historical range of radiographic modalities [3]. Notably, PPD alone was insufficient in several cases: Three NL-ARM cases with PPD ≤ 10 mm were correctly classified as NL-ARM only because of meconium discharge through the urinary tract, and one L-ARM case with a PPD of 13.0 mm on postnatal day 0 was misclassified as NL-ARM, resulting in an unnecessary colostomy. This misclassified case underscores the dynamic nature of the pouch position in the early neonatal period. The inadequate timing of ultrasonographic assessment likely contributed to the incomplete pouch descent and overestimation of PPD, a well-recognized pitfall of day-0 ultrasonographic assessment. Based on this experience, our current institutional practice has shifted toward repeated PPD measurements, particularly on postnatal day 1 and, when necessary, immediately before surgery, to account for physiological changes. This modification was specifically implemented to capture dynamic changes in pouch descent, a factor that previous studies and our own dataset have shown to influence PPD measurement accuracy substantially. This adjustment reflects the inherent strengths and limitations of PPD measurements and reinforces the need for multimodal evaluations. When PPD findings were integrated with clinical and radiological assessments, diagnostic accuracy improved substantially, supporting the robustness of our multimodal diagnostic algorithm for early ARM classification [7, 8].

### Limitations

This study had several limitations. First, its retrospective design and inclusion of patients from two tertiary referral centers may have introduced selection bias. Second, although the 10-mm PPD cut-off showed excellent discriminative performance in our cohort, it may not be directly generalizable to other settings because of potential interobserver variability and differences in measurement technique, ultrasonographic equipment, and timing of assessment. Third, the ROC analysis was based on a relatively small subgroup of patients without visible perineal fistulas, and the resulting high AUC may have overestimated the true discriminative performance of PPD. Fourth, the reported ROC-based performance of PPD was derived from all 51 patients without visible perineal fistulas and does not directly represent the performance of PPD when used as the final classification criterion within the multimodal algorithm, which applied to a more selected subgroup. Finally, intraoperative confirmation of ARM subtype was not blinded to the preoperative ultrasonographic findings, which may have introduced assessment bias. Further multicenter prospective studies with standardized imaging protocols and blinded diagnostic evaluation are needed to validate and refine this multimodal diagnostic algorithm.

## Conclusion

In summary, ultrasonographic PPD measurement is a useful adjunct for early neonatal ARM assessment, but its value is maximized when integrated with physical examination, meconium passage assessment, and contrast studies. This multimodal strategy achieved high diagnostic accuracy and may help avoid unnecessary colostomy in selected patients.

## Data Availability

The datasets generated and/or analyzed during the current study are not publicly available because they contain clinical data from individual patients but are available from the corresponding author on reasonable request.
